# Laparoscopic left hemi-nephroureterectomy for a patient with suspected urothelial carcinoma in a horseshoe kidney

**DOI:** 10.1093/jscr/rjac025

**Published:** 2022-03-02

**Authors:** Orla Cullivan, Kevin Byrnes, Frank D’Arcy

**Affiliations:** Department of Urology, Galway University Hospital, Galway H91YR71, Ireland

## Abstract

A 76-year-old gentleman with a background of benign prostatic hyperplasia, hiatus hernia and anxiety was referred to the rapid access haematuria service following an episode of painless visible haematuria. Flexible cystoscopy did not reveal any concerning bladder lesions. CT Urogram demonstrated a horseshoe kidney with a filling defect in the left upper pole moiety suspicious for an urothelial carcinoma. The patient was subsequently referred to the urology services in a tertiary centre. Flexible ureterorenoscopy was performed, with findings of a likely urothelial carcinoma corresponding to the suspicious area on imaging. Biopsy of this lesion revealed a low grade urothelial cancer. The patient proceeded to have a laparoscopic left heminephroureterectomy with an open bladder cuff. The patient recovered well and urinary catheter was removed Day 12 post procedure after the performance of a cystogram. Histology revealed a favourable pTa low grade malignancy, and the patient will require ongoing follow-up moving forward. This case report highlights the operative intricacies in managing patients with horseshoe kidney due to anatomic variations associated with this condition.

## INTRODUCTION

Horseshoe kidney represents one of the most common congenital renal abnormalities, with an estimated incidence in 1:500. Due to a number of anatomical factors, horseshoe kidneys pose an operative challenge for surgeons. The following case report explores treatment for a left-sided urothelial carcinoma in a patient with a horseshoe kidney via a laparoscopic nephroureterectomy.

## CASE PRESENTATION

**Figure 1:**
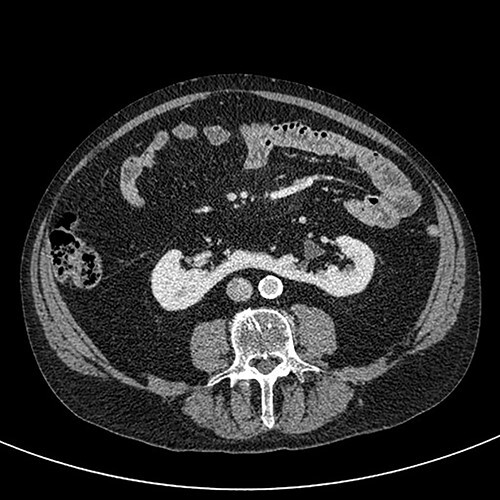
Axial CT (nephrographic phase).

**Figure 2:**
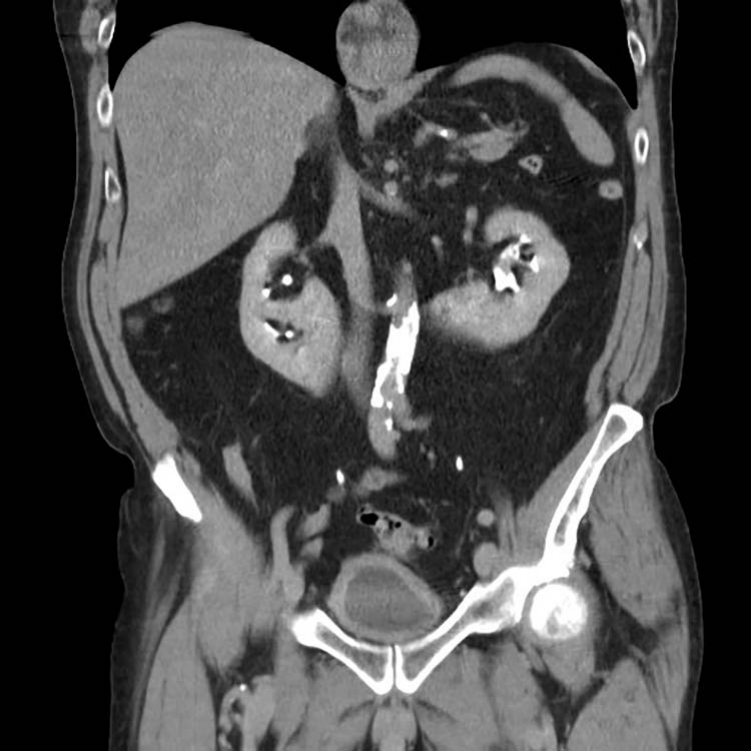
Coronal CT (urographic phase) demonstrating filling defect left upper pole.

A 76-year-old gentleman with a background of benign prostatic hyperplasia, hiatus hernia, and anxiety was referred to the rapid access haematuria service following an episode of painless visible haematuria. Flexible cystoscopy did not reveal any concerning bladder lesions. CT Urogram demonstrated a horseshoe kidney with a filling defect in the left upper pole moiety suspicious for an urothelial carcinoma (Figs 1 and 2). The patient was subsequently referred to the urology services in a tertiary centre. Flexible ureterorenoscopy was performed, with findings of a likely urothelial carcinoma corresponding to the suspicious area on imaging. Biopsy of this lesion revealed a low grade urothelial cancer.

The patient proceeded to have a laparoscopic left heminephroureterectomy with an open bladder cuff. Under general anaesthesia, the patient was placed in the right lateral decubitus position, with access obtained via a 15 mm supraumbilical incision (Hassan technique). Further ports were placed under laparoscopic vision. The descending colon was mobilized and the spleen dropped. Ureter and isthmus were identified. Hilum was dissected. The left moiety had two arteries and two veins, each of which was secured with a haemalock. The isthmus was flattened at the narrowest point and then divided with a purple EndoGIA. The ureter was tracked to the bladder and a formal bladder cuff performed, with a two-layer closure of the bladder. Estimated blood loss was 500 ml.

Postoperatively, the patient had a temperature spike on postop Day 1, and a sepsis screen was performed. Chest radiograph revealed left basal atelectasis, which was treated with a week of antibiotics. The patient recovered well, and his urinary catheter was removed Day 12 post procedure after the performance of a cystogram demonstrating no bladder leak. Histology revealed a favourable pTa low grade malignancy, and the patient will require biannual cystoscopies and CT imaging as follow-up.

## DISCUSSION

Horseshoe kidney is the most common fusion defect of the kidney, with an incidence of approximately 1 in 500, and a 2:1 male preponderance [1]. A horseshoe kidney results from fusion of both kidneys, usually at the lower poles, joined together by a fibrous or parenchymal isthmus [2]. These patients can be prone to a variety of complications including renal tract calculi, pelviuretric junction obstruction and renal tumours [1]. The incidence of urothelial carcinoma is higher in patients with horseshoe kidney, likely due to factors such as chronic obstruction, recurrent urinary tract infections and stone formation [2]. Nevertheless, renal cell carcinoma remains the most common renal cancer in horseshoe kidney patients.

There are a number of anatomic factors associated with horseshoe kidney that pose an operative challenge during nephrectomy. These include a more inferior location of the kidney, with 40% located beneath the inferior mesenteric artery and 20% located in the pelvis, as well as renal malrotation with consequent abberancy in ureteric anatomy [3]. In addition, horseshoe kidneys have highly variable vasculature, with single or multiple renal arteries potentially arising from the aorta, iliac arteries or inferior mesenteric artery [4]. In one study, horseshoe kidneys had nearly double the amount of supplying vessels when compared with normal kidneys, with frequent instances of renal arteries entering directly into the renal parenchyma as opposed to via the hilum [5]. In 80% of cases, the isthmus contains functional renal tissue and has a variable blood supply, which can make dissection at surgery difficult [3].

Upper tract urothelial carcinoma represents 5–7% of all renal tumours, and operative treatment with nephroureterectomy is often required for these patients [6]. Minimally invasive surgery via the transperitoneal route is an acceptable treatment pathway in such cases, though the existing literature is sparse [7]. Due to the aforementioned anatomical variations associated with horseshoe kidneys, particularly their vasculature and limited mobilization, meticulous preoperative planning is essential [8]. Potential complications of laparoscopic nephrectomy in patients with horseshoe kidney include conversion to open, major bleeding and damage to other organs [9].

Although urothelial carcinoma in the context of a patient with a horseshoe kidney represents an uncommon presentation, it does pose a major operative challenge for the surgeon. Consequentially, thorough preoperative assessment and planning, and appropriate selection of operative approach are imperative.

## CONFLICT OF INTEREST STATEMENT

None declared.

## FUNDING

None.
